# High serine:glyoxylate aminotransferase activity lowers leaf daytime serine levels, inducing the phosphoserine pathway in Arabidopsis

**DOI:** 10.1093/jxb/erw467

**Published:** 2016-12-23

**Authors:** Katharina Modde, Stefan Timm, Alexandra Florian, Klaudia Michl, Alisdair R. Fernie, Hermann Bauwe

**Affiliations:** 1 University of Rostock, Plant Physiology Department, Albert-Einstein-Straße 3, D-18051 Rostock, Germany; 2 Max Planck Institute of Molecular Plant Physiology, Am Mühlenberg 1, D-14476 Golm, Germany

**Keywords:** *Arabidopsis thaliana*, phosphoserine pathway, photorespiration, serine, serine biosynthesis, serine:glyoxylate aminotransferase.

## Abstract

Serine:glyoxylate aminotransferase (SGAT) converts glyoxylate and serine to glycine and hydroxypyruvate during photorespiration. Besides this, SGAT operates with several other substrates including asparagine. The impact of this enzymatic promiscuity on plant metabolism, particularly photorespiration and serine biosynthesis, is poorly understood. We found that elevated SGAT activity causes surprisingly clear changes in metabolism and interferes with photosynthetic CO_2_ uptake and biomass accumulation of Arabidopsis. The faster serine turnover during photorespiration progressively lowers day-time leaf serine contents and in turn induces the phosphoserine pathway. Transcriptional upregulation of this additional route of serine biosynthesis occurs already during the day but particularly at night, efficiently counteracting night-time serine depletion. Additionally, higher SGAT activity results in an increased use of asparagine as the external donor of amino groups to the photorespiratory pathway but does not alter leaf asparagine content at night. These results suggest leaf SGAT activity needs to be dynamically adjusted to ensure (i) variable flux through the photorespiratory pathway at a minimal consumption of asparagine and (ii) adequate serine levels for other cellular metabolism.

## Highlight

A promiscuous photorespiratory aminotransferase contributes to leaf serine homeostasis, guiding serine biosynthesis at night, and diverts asparagine from other cellular processes into photorespiration.

## Introduction

**Fig. 1.**
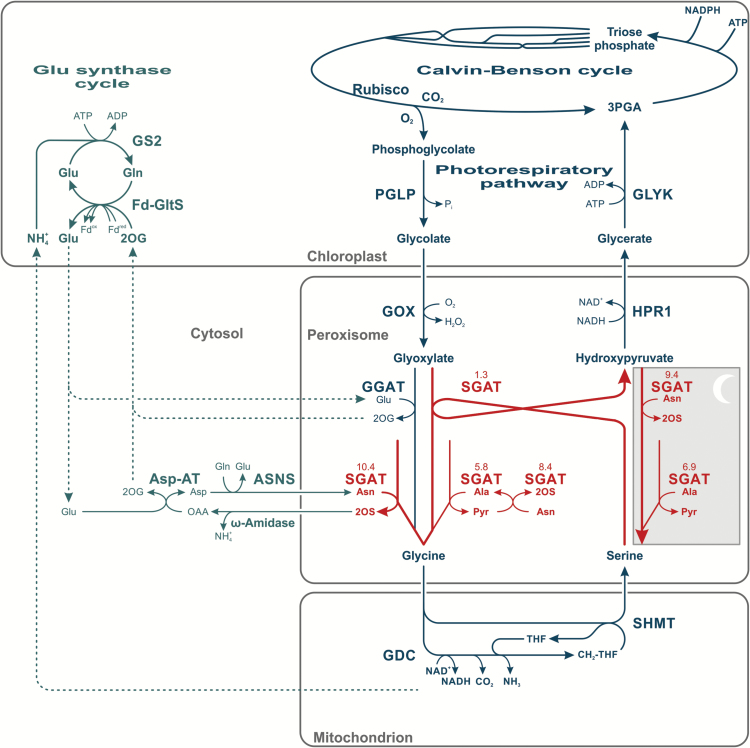
Impact of excess SGAT activity on the photorespiratory pathway and photorespiratory nitrogen cycling. Red arrows indicate excess ‘SGAT’ activities including entry nodes for asparagine nitrogen. Red numbers are catalytic efficiencies of the individual ‘SGAT’ reactions according to Zhang *et al.* (2013). Also shown are potential serine production from hydroxypyruvate at night (grey background) and the recycling of 2OS via ω-amidase at day and night. Abbreviations: 2OG, 2-oxoglutarate; 2OS, 2-oxosuccinamate; Asp-AT, aspartate aminotransferase; ASNS, asparagine synthetase; Fd-GltS, glutamate synthase; GDC, glycine decarboxylase; GGAT, glutamate:glyoxylate aminotransferase; GLYK, glycerate 3-kinase; GOX, glycolate oxidase; GS2, glutamine synthetase; HPR1, hydroxypyruvate reductase; PGLP, phosphoglycolate phosphatase; SHMT, serine hydroxymethyltransferase.

Photorespiration is an essential metabolite repair and salvage pathway of all plants. It serves to recycle 2-phosphoglycolate (2PG), which is produced as a side-product of oxygenic photosynthesis, into 3-phosphoglycerate (3PGA; Fig. 1). The actual conversion of the glycolate two-carbon skeleton into a three-carbon skeleton that is compatible with the Calvin–Benson cycle requires oxidation to glyoxylate followed by transamination to glycine. This amino acid has a very central position in the photorespiratory pathway because it can serve as a one-carbon donor (in the glycine decarboxylase (GDC) reaction, releasing one molecule each of CO_2_ and NH_3_) and as a one-carbon acceptor (in the serine hydroxymethyltransferase (SHMT) reaction, producing serine). Serine:glyoxylate aminotransferase (SGAT; EC 2.6.1.45) then returns the serine amino group back to glyoxylate, providing a fresh molecule of glycine and hydroxypyruvate for the enzyme hydroxypyruvate reductase 1 (HPR1). The ammonia released during the oxidative decarboxylation of glycine is re-fixed in the glutamate synthase cycle first in glutamine and then in glutamate, which is subsequently used by glutamate:glyoxylate aminotransferase (GGAT; EC 2.6.1.4) to form the second glycine needed for serine production in the combined GDC–SHMT reaction. Whilst the recycling of 2PG to 3PGA is the primary and most important function of the photorespiratory pathway, it is also relevant for stress protection (Wingler *et al.*, 2000) and the predominant source of glycine and serine in photosynthesizing organs (Ros *et al.*, 2014). Both amino acids are essential for many further key metabolic processes. Additionally, serine is involved in the transcriptional regulation of photorespiration (Timm *et al.*, 2013) and the induction of serine biosynthesis in mammals (Ye *et al.*, 2012; Häusler *et al.*, 2014).

A large number of studies have shown that photosynthetic carbon assimilation becomes impaired when carbon or nitrogen flow through the photorespiratory pathway is artificially restricted (reviewed in Timm and Bauwe, 2013). This also holds for the photorespiratory aminotransferases (Somerville and Ogren, 1980; Murray *et al.*, 1987; Havir and McHale, 1988; McHale *et al.*, 1988; Wingler *et al.*, 1999*a*; Igarashi *et al.*, 2003; Liepman and Olsen, 2001, 2003; Dellero *et al.*, 2015). By contrast, in the context of crop breeding, it is remarkable that transgenic plants overexpressing photorespiratory enzymes displayed higher photosynthetic efficiency and better growth (GDC-H and GDC-L in Arabidopsis, Timm *et al.*, 2012*a*, 2015; SHMT1 in rice, Wu *et al.*, 2015), a broader optimum growth temperature (HPR1 in tobacco, Oliver *et al.*, 1995), or improved salt stress tolerance (SGAT in duckweed, Yang *et al.*, 2013). It is not yet exactly known how these enhancements are achieved on the molecular level and to what extent this includes crosstalk within the photosynthetic–photorespiratory metabolic network, with other metabolism, and with gene expression.

The introductory rough draft of photorespiratory metabolism presumes that the two branches of the photorespiratory pathway are statically coupled via SGAT and, secondly, that glutamate nitrogen is the exclusive extra-peroxisomal source from which ammonia losses from the core pathway are replenished and additional amino groups provided to support flux whenever necessary during the day. Both abstractions are useful but also simplifying. Particularly, they do not consider that the two canonical photorespiratory aminotransferases are promiscuous enzymes that transaminate a range of different substrates (Rehfeld and Tolbert, 1972; Ireland and Joy, 1983; Murray *et al.*, 1987; Liepman and Olsen, 2001, 2003; Kendziorek and Paszkowski, 2008; Zhang *et al.*, 2013). SGAT, on which this report is focused, actually works most efficiently with asparagine as the amino donor (EC 2.6.1.14) to convert pyruvate, hydroxypyruvate and glyoxylate into the corresponding amino acids (Zhang *et al.*, 2013). The 2-oxosuccinamate (2OS) produced is hydrolysed to oxaloacetate and ammonia by ω-amidase (Streeter, 1977; Zhang and Marsolais, 2014). Such a link, in which asparagine would serve as an additional amino donor for photorespiration, was proposed years ago (for example Ta *et al.*, 1985), whereas more recent data suggest asparagine normally does not contribute to the photorespiratory nitrogen cycle but rather has a specific role in phloem loading and nitrogen reallocation (Gaufichon *et al.*, 2013, 2016). This conflict is not yet resolved.

In this study, we overexpressed *SGAT* in Arabidopsis leaves with three main aims: (i) to examine the effect of this intervention on photosynthesis and growth, (ii) to test the suggested function of serine for the regulation of transcription and metabolism under *in planta* low-serine conditions, and (iii) to examine the controversially discussed role of asparagine for photorespiratory metabolism.

## Materials and methods

### Plant materials and plant growth

*Arabidopsis thaliana* (Arabidopsis) ecotype Col-0 was the wild type reference and used for genetic transformation. Seeds from the same harvest of the wild type and three stable T3 lines selected according to leaf SGAT activities (wild type<L1<L2<L3; Supplementary Fig. S1 at *JXB* online) were surface sterilized with hypochlorous acid, incubated at 4 °C for at least 2 d to break dormancy, and germinated on soil for growth to stage 5.1 (fully developed rosette leaves; Boyes *et al.*, 2001) or on half-strength MS medium supplemented with 1% sucrose for growth to stage 1.04 (four rosette leaves >1 mm). All plants were grown under controlled environment with a day/night cycle of 10/14 h at 20/18 °C, ~120 µmol m^–2^ s^–1^ irradiance, 390 µL L^–1^ CO_2_, and 70% relative humidity. For soil cultures, we used a 4:1 mixture of Mini Tray (Einheitserdewerk, Uetersen) and vermiculite regularly watered with 0.2% Wuxal liquid fertilizer (Aglukon).

### cDNA cloning and gene expression

The entire *SGAT* coding sequence (1206 bp) was PCR-amplified from *Flaveria pringlei* cDNA using the primers *FpSGAT*-cDS-fw (sense) and *FpSGAT*-cDS-rev (antisense), introducing terminal restriction sites for *Bam*HI and *Xma*I. For oligonucleotide sequences see Supplementary Table S1. The amplificate was ligated into the pGEM-T vector (Invitrogen) and sequenced. Next, the *Bam*HI–*Xma*I fragment was excised and ligated in front of the CaMV polyA site of the pGreen 35S-CaMV cassette (http://www.pgreen.ac.uk/) to generate *FpSGAT:CaMV*, which then was excised via *Xba*I and *Eco*RV and ligated into the binary plant transformation vector pGREEN0229 containing the light-inducible *ST*-*LS1* promoter used previously (Stockhaus *et al.*, 1989; Timm *et al.*, 2012*a*). This construct (see Supplementary Fig. S1A) was introduced into *Agrobacterium tumefaciens* strain GV3101 and used to genetically transform Arabidopsis Col-0 (Clough and Bent, 1998). Phosphinotricine (Basta)-resistant progeny of three independent transformation events were selected according to leaf *FpSGAT* transcript contents and used to generate stable T3 lines (L1, L2, and L3) for all further experiments.

### Verification of transgenic lines, RT-PCR and qRT-PCR

To verify the integration of the *FpSGAT* overexpression construct, leaf DNA was PCR-amplified with primers specific for the *FpSGAT* fragment (1 min at 94°C, 1 min at 58°C, 1.5 min at 72°C; 35 cycles) and the *S16* gene (1 min at 94°C, 1 min at 58°C, 30 s at 72°C; 35 cycles), which encodes a 40S ribosomal protein and served as a loading control. Expression of the *FpSGAT* transgene was verified by RT-PCR, using 2.5 µg of leaf RNA for cDNA synthesis (Nucleospin RNA plant kit, Macherey-Nagel, and RevertAid cDNA synthesis kit, MBI Fermentas) and the primer combination *FpSGAT*-cDS-fw and *FpSGAT*-cDS-rev, yielding a 1206 bp PCR product for the full-length *FpSGAT* transcript.

Expression of genes encoding enzymes of photorespiration and the phosphoserine pathway was quantified by real-time PCR using cDNA prepared from stage 1.04 seedlings or stage 5.1 adult plants and a light cycler 1.5 system (Roche, Grenzach-Wyhlen, Germany) with SYBR Green fluorescence (Roche, Germany) in six biological and three technical replicates as described previously (Timm *et al.*, 2013). Signals corresponding to the 432 bp amplificate of the constitutively expressed gene *S16* (sense primer *S16*-fw-RT, antisense primer *S16*-rev-RT) were used for calibration.

### Protein isolation and enzyme activities

Leaf protein was extracted from 100 mg tissue in 200 µL of pre-cooled buffer (25 mM HEPES-KOH, 1 mM EDTA, 1 mM MgCl_2_, 1 mM KCl, 10 mM β-mercaptoethanol, 0.1 mM phenylmethylsulfonyl fluoride, 10% glycerol, pH 7.6). After 10 min centrifugation (20 000 *g*, 4 °C), protein concentrations were determined (Bradford, 1976). Activities of SGAT were measured according to Liepman and Olsen (2001) and activities of hydroxypyruvate and glyoxylate reductases as described in Timm *et al.* (2008).

### Gas exchange

Gas exchange was measured using fully expanded rosette leaves of plants at growth stage 5.1 as described previously (Timm *et al.*, 2011) under the following conditions: photon flux density 1000 µmol m^–2^ s^–1^, temperature 25 °C, flow rate 300 µmol s^–1^, relative humidity ~70%. Percentage inhibition of CO_2_ assimilation (*A*) was calculated from measurements at 21 and 40% O_2_ as follows: O_2_-inhibition=100×(A_21_–A_40_)/A_21_. γ values were obtained by linear regression of the Γ *versus* % oxygen concentration response as slopes of the regression lines.

### Growth and biomass

Representative photographs were taken from transgenic lines and the wild type grown side-by-side under standard conditions. Rosette areas were determined at weeks 4–8 after germination. Every week, six individual plants were photographed at the middle of the day and areas quantified using ImageJ software (http://rsb.info.nih.gov/ij/). At stage 5.1, total leaf numbers were counted, leaf rosette diameters determined as the longest possible distance, and rosette fresh and dry weights measured.

### Metabolite analysis

Amino acids were quantified in leaf samples of stage 5.1 plants harvested at the end of the day (EoD; after 9 h illumination) and end of the night (EoN; after 13 h darkness). The analytical procedure is described elsewhere (Hagemann *et al.*, 2005). Other metabolites were quantified by gas chromatography coupled to mass spectrometry (GC-MS) analysis of leaf samples of stage 5.1 plants harvested at EoD and EoN and processed as recommended in Fernie *et al.* (2011) and Lisec *et al.* (2006). Five biological replicates per genotype were analysed for each data set.

### Statistical analysis

Statistical tests were performed using Student’s two tailed *t*-test (Microsoft Excel 10.0).

### Accession numbers

Sequence data from this article can be found in the Arabidopsis Genome Initiative database under the following accession numbers: At2g13360 (SGAT), At1g11860 (GDC-T), At4g33010 (GDC-P), At4g37930 (SHM1), At1g68010 (HPR1), At1g80380 (GLYK), At5g40760 (cG6PD), At5g36700 (PGLP), At1g18640 (PSP), At4g35630 (PSAT), At1g17745 (PGDH), and At2g09990 (40S ribosomal protein S16).

## Results

### Overexpression lines and SGAT activities

**Fig. 2.**
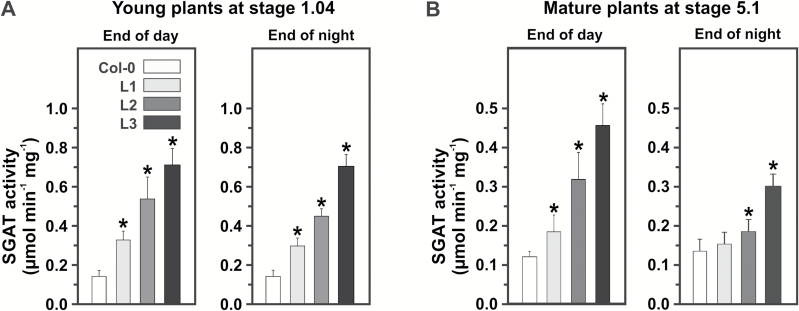
Leaf SGAT activities in the wild type and in three transgenic lines. Enzyme activities were measured in rosette leaves from (A) seedlings at stage 1.04 according to Boyes *et al.* (2001) and (B) mature plants at stage 5.1 at the end of the day and end of the night. Values are means±SD from four biological replicates. Asterisks indicate that the difference from the wild-type control is significant based on Student’s *t*-test (*P<0.05).

For *SGAT* overexpression in Arabidopsis, we used a *Flaveria pringlei* SGAT (*FpSGAT*) cDNA in combination with the potato *ST*-*LS1* promoter (Stockhaus *et al.*, 1989) and the cauliflower mosaic virus terminator (see Supplementary Fig. S1A). The homologous *Flaveria* cDNA was chosen to reduce interference with endogenous *SGAT* transcripts and this particular promoter used because it confers high light-inducible gene expression only in chloroplast-containing cells. Three transgenic lines, L1, L2 and L3, were selected and propagated to yield stable T_3_ generations. Leaf SGAT activity was up to five-fold elevated (L1, 2.3-fold; L2, 3.8-fold; L3, 5-fold; Fig. 2A) in MS-grown seedlings at growth stage 1.04 (Boyes *et al.*, 2001). Approximately corresponding significant increases were also observed in leaves of all three lines grown in soil to stage 5.1 (Fig. 2B).

### Photosynthetic CO_2_ uptake is reduced in SGAT overexpressors

**Fig. 3.**
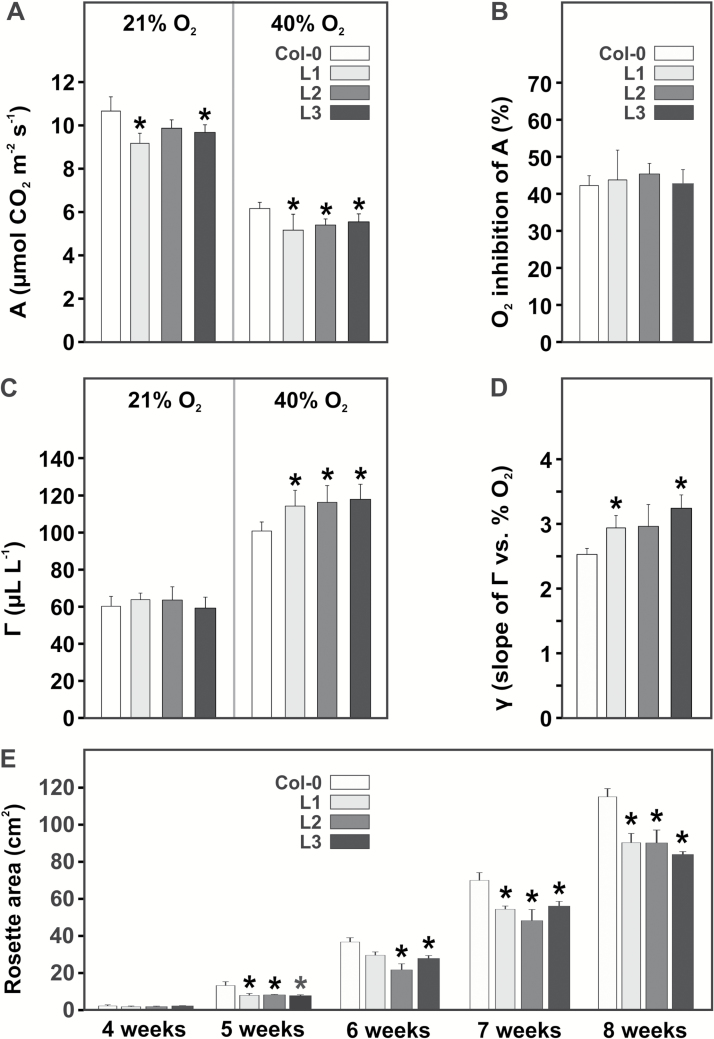
Photosynthetic parameters of *SGAT* overexpressor leaves at growth stage 5.1 and growth over 8 weeks. (A) Net CO_2_ uptake rates (*A*) at 390 µL L^–1^ CO_2_, 21% and 40% O_2_. (B) CO_2_ compensation points Γ at 21% and 40% oxygen. (C) Oxygen inhibition of *A* and (D) oxygen response of Γ (γ). Values for A–D are means±SD from at least four biological replicates. Asterisks indicate significant differences from the wild type based on Student’s *t*-test (*P*<0.05; n.s., not significant). (E) Leaf areas at five time points after germination. Values given are means±SD (*n* = 6). Asterisks indicate significant differences from the wild type at the respective time point based on Student’s *t*-test (*P<0.05).

The impact of overexpressing *SGAT* on photosynthetic performance was determined by measuring several gas-exchange parameters at two oxygen concentrations, 21% and 40%. As shown in Fig. 3A–D, the overexpressor lines display reduced net CO_2_ uptake rates at air CO_2_ concentrations (*A*; significant in lines 1 and 3 at 21% O_2_ and in all transgenic lines at 40% O_2_). The calculated oxygen inhibition of *A* however is not clearly different between the genotypes. CO_2_ compensation points (Γ) remain on the wild-type level at 21% O_2_ but are higher (significant in lines L1 and L3) than the wild-type value at 40% O_2_. All transgenic lines correspondingly display a significantly stronger response of Γ to changes in the O_2_ concentration than the wild type, indicating that the extent to which SGAT activity interferes with photosynthesis increases at higher photorespiratory flux but altogether has no very large effect on photosynthetic CO_2_ fixation.

### SGAT overexpressors show altered leaf numbers and leaf expansion

**Fig. 4.**
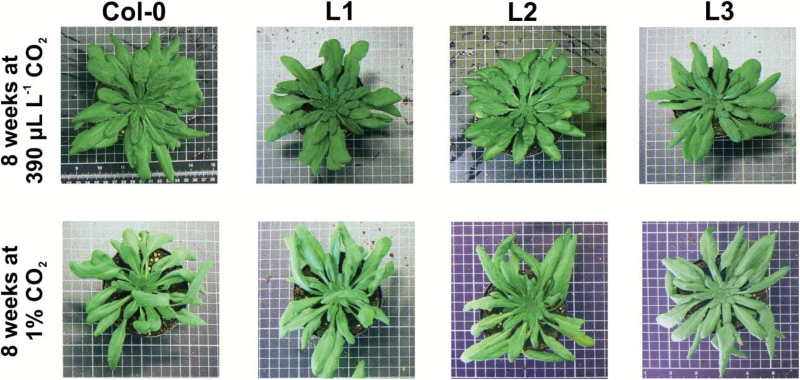
Representative photographs of *SGAT* overexpressor plants and the wild type grown in normal air and air with 1% CO_2_. Plants were grown under a photoperiod of 10/14 h and 20/18 °C in the day/night cycle, 75% relative humidity, at a light intensity of ~120 µmol photons m^–2^ s^–1^ for 8 weeks in normal air (about 390 µL L^–1^ CO_2_) and in 1% CO_2_, respectively.

Over the life cycle of Arabidopsis, the reduced photosynthetic performance of the overexpressor lines is accompanied by an altered phenotype under standard conditions as visible in smaller leaf numbers, reduced leaf expansion, and less fresh weight relative to the wild type (Table 1 and Fig. 4). If grown for 8 weeks under standard conditions of a 10/14 h day/night cycle at 390 µL L^–1^ CO_2_, the transgenic lines developed up to 25% fewer leaves, displayed smaller rosette areas by about 20–25%, and accumulated 8–15% less fresh weight compared with the side-by-side grown wild type. Onset of flowering was unchanged. Smaller rosette areas in comparison with the wild type were noticeable as from the fourth week after germination (Fig. 3E). None of these growth-related alterations was detectable if the plants were grown in air enriched with 1% CO_2_, which strongly inhibits photorespiration (Table 1). Lower biomass accumulation under long-term high CO_2_ exposure is due to a number of factors including lower stomatal conductance (Lammertsma *et al.*, 2011), downregulation of photosynthesis (Stitt and Krapp, 1999; Long *et al.*, 2004) and/or impaired nitrate assimilation (Bloom, 2015). Taken together, these data demonstrate that the reduced growth of *SGAT* overexpressors in air is in the greater part due to a perturbation of photorespiration and possibly of associated pathways with leaf nitrogen metabolism as a prime candidate.

**Table 1.** SGAT overexpressors grow slower than the wild type in normal air but wild-type-like at 1% CO_2_ Plant were grown to stage 5.1 (Boyes *et al.*, 2001). Values are mean±SD from at least six biological replicates per genotype. Numbers marked with an asterisk are statistically significant from the wild type based on Student’s *t*-test (*P < 0.05).

Growth parameter	Col-0	L1	L2	L3
Normal air (0.039% CO_2_)				
Diameter (cm)	15.4 ± 1.37	15.1 ± 1.76	16.2 ± 2.20	14.8 ± 2.23
Leaf number	62.3 ± 3.01	52.5 ± 2.74*	53.0 ± 6.42*	46.2 ± 4.49*
Rosette area (cm^–2^)	115 ± 4.45	90.3 ± 4.99*	90.2 ± 6.93*	84.0 ± 1.53*
Fresh weight (g)	5.79 ± 0.63	5.03 ± 0.35*	5.00 ± 0.60*	5.14 ± 0.22*
Dry weight (g)	0.49 ± 0.05	0.42 ± 0.06	0.44 ± 0.04	0.48 ± 0.04
FW/DW ratio	12.1 ± 0.61	11.4 ± 0.95	11.2 ± 0.63	10.7 ± 1.12*
Elevated CO_2_ (1% CO_2_)				
Diameter (cm)	14.9 ± 1.51	14.9 ± 1.36	14.5 ± 1.04	13.2 ± 2.14
Leaf number	32.8 ± 6.52	36.2 ± 4.36	36.5 ± 2.89	36.5 ± 6.12
Rosette area (cm^–2^)	206 ± 15.2	222 ± 10.9	215 ± 8.48	209 ± 4.37
Fresh weight (g)	2.83 ± 0.87	3.08 ± 0.61	2.88 ± 0.39	2.35 ± 0.87
Dry weight (g)	0.23 ± 0.06	0.27 ± 0.06	0.28 ± 0.04	0.30 ± 0.07
FW/DW ratio	11.7 ± 2.89	11.8 ± 3.69	10.5 ± 1.36	9.44 ± 0.48

### Leaf serine and asparagine contents are lower during the day but not at night

**Fig. 5.**
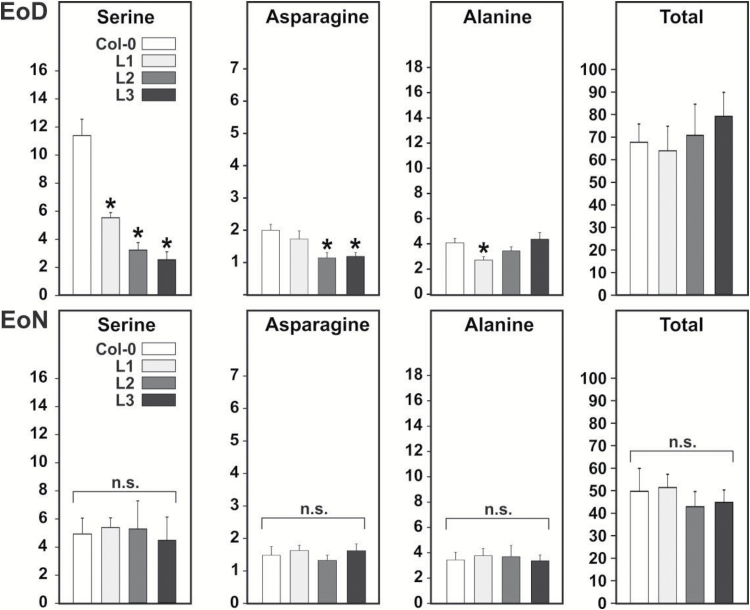
Day and night leaf amino acid contents at growth stage 5.1. Rosette leaf samples were harvested after 9 h illumination (EoD) and after 13 h darkness (EoN). Values are means±SD (*n*>4) in µmol g^–1^ fresh weight. Asterisks indicate significant differences from the wild type based on Student’s *t*-test (*P < 0.05; n.s., not significant). For the complete list see Supplementary Tables S1 and S2; high-CO_2_ data are shown in Supplementary Table S3.

In order to find out whether the negative effect of *SGAT* overexpression on growth is related to a suboptimal nitrogen flow through photorespiration, possibly due to increased import of asparagine amino groups, we quantified the steady-state pools of soluble amino acids in mature leaves harvested at the end of the day (EoD) and the end of the night (EoN) by high-performance liquid chromatography (HPLC; Fig. 5, absolute values in Supplementary Tables S1 and S2). At EoD, asparagine contents were reduced by about 40–50% (significant in lines L2 and L3) and serine contents by about 50–75% (significant in all transgenic lines). At EoN, serine and asparagine contents were not markedly different from the wild-type values in mature leaves. Interestingly, EoD levels of glutamine (by up to 25%, significant only in L3) and glutamate (by up to 90%, significant in all transgenic lines) were increased (see Supplementary Table S2). This result could correspond to the opinion that these two amino acids monitor ammonia production relative to 2-oxoglutarate synthesis (Novitskaya *et al.*, 2002): two instead of one ammonia molecules would be released per asparagine used in photorespiration (see Fig. 1). In fact, daytime Glu (and the Glu/Gln ratio) could potentially increase as the result of higher Asn requirements for SGAT (10.4, compare Fig. 1) and in turn readjustment of the glutamate synthase cycle. This interpretation is purely hypothetical though. The levels of alanine, which is (Betsche, 1983; Ta and Joy, 1986; Zhang *et al.*, 2013) or is not (Rehfeld and Tolbert, 1972) considered as a potential donor of amino groups to the photorespiratory pathway, and most other amino acids were essentially unaltered or not consistently altered in the day and night. Plants grown to stage 5.1 under the non-photorespiratory condition of 1% CO_2_ (Supplementary Table S3) did not display significant differences between the transgenic lines and the wild type in the contents of most amino acids including asparagine and serine. The only exception was a decrease in methionine under non-photorespiratory conditions (down to 25%, significant in L2 and L3 grown in air at EoN, Supplementary Table S4; down to 30%, significant in all three transgenic lines grown under 1% CO_2_ at EoD, Supplementary Table S3). The lower contents of this amino acid are likely related to the role of serine for the biosynthesis of cysteine and subsequently methionine (Takahashi *et al.*, 2011). The normalizing effects of high CO_2_ altogether suggest that metabolic changes induced by *SGAT* overexpression are most distinct at high photorespiratory flux but to some extent also visible in a non-photorespiratory environment.

### Other main metabolic routes are essentially unaltered in the daytime

**Fig. 6.**
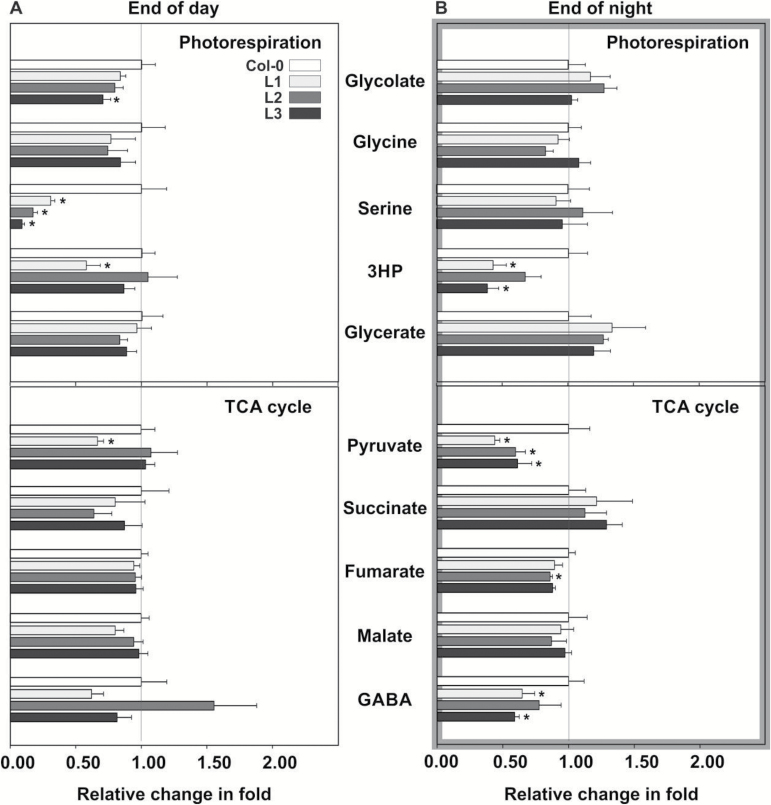
Relative leaf contents of selected metabolites at growth stage 5.1. Leaf samples were harvested (A) after 9 h illumination at the end of the day and (B) after 13 h darkness at the end of the night. Fold values are means±SD of the respective GC-MS data from at least four biological replicates. Asterisks indicate that the difference from the wild type control is significant based on Student’s *t*-test (*P < 0.05). The complete list is provided as Supplementary Table S5. Abbreviations: 3HP, hydroxypyruvate; GABA, γ-aminobutyric acid.

We next examined relative leaf metabolite contents of air-grown stage 5.1 plants sampled at EoD and EoN by gas chromatography coupled to mass spectrometry (GC-MS). These data (Fig. 6, full set in Supplementary Table S5) confirmed the significantly lower daytime contents of serine and asparagine and additionally showed that most other metabolites were only marginally or not consistently altered. EoD glycolate contents were somewhat lower (significant only in line L3), corresponding to the slower net CO_2_ uptake shown in Fig. 3. The contents of several TCA cycle metabolites (citrate, succinate, fumarate and malate) were essentially unchanged in comparison with the wild type in the day and night, whereas that of 2-oxoglutarate (2OG) was unaltered at EoD but significantly reduced in all lines at EoN. Moreover, the TCA cycle-associated metabolite γ-aminobutyrate (GABA) was unaltered in all three lines at EoD but significantly lower in lines L1 and L3 at EoN. Notably, we also observed a distinct drop in the steady-state content of hydroxypyruvate at EoN (significant in L1 and L3; Fig. 6) that did not consistently occur at EoD. This drop, in combination with the wild-type-like re-normalized EoN serine contents in the overexpressor plants (Fig. 5A), could indicate that the low daytime serine levels in some way trigger operation of the glycerate pathway of serine synthesis at night.

### Activities of enzymes for glycerate oxidation are higher at night

Enhanced consumption of hydroxypyruvate for serine synthesis at night would also require enhanced re-synthesis from glycerate via D-glycerate dehydrogenase, which is one step in the glycerate pathway of serine synthesis. The thermodynamic equilibrium of this reaction favours glycerate production, and the reverse reaction towards hydroxypyruvate formation is about 30-fold slower (Kohn and Warren, 1970). We instead measured glycerate production, and we also used glyoxylate as a substrate because of possible overlap with glyoxylate reductases (discussed in Allan *et al.*, 2009; Givan and Kleczkowski, 1992). These data are shown in Table 2. At EoD, there were no significant differences between *SGAT* overexpressors and the wild type for any of the used substrate combinations. At EoN, there was an up to 19% (not significant) increase in the NADH-dependent HPR activity (essentially the peroxisomal HPR1) and a two-fold significant increase in the NADPH-dependent HPR activity (mostly due to the cytosolic enzyme hydroxypyruvate reductase 2, HPR2) in all transgenic lines while the glyoxylate-dependent activities remained unaltered. In absolute numbers, the 19% non-significant increase in ‘HPR1’ activity alone would likely provide more additional glycerate dehydrogenase activity in the *SGAT* overexpressors at night than the doubled ‘HPR2’ activity.

**Table 2.** Glycerate dehydrogenase activities in leaf extracts of SGAT overexpressors Soluble leaf proteins were extracted from rosette leaf samples harvested after 9 h illumination (EoD) and 13 h darkness (EoN) in the day/night cycle at stage 5.1 (Boyes *et al.*, 2001). Assays were performed in the oxoacid-reducing direction. Abbreviations: GR, glyoxylate reductase; HPR, hydroxypyruvate reductase. Values are means ±SD in nmol min^–1^ mg^–1^ protein with the respective oxoacid and the indicated coenzyme from at least five biological replicates per genotype. Numbers marked with an asterisk are statistically significant from the wild type based on Student’s *t*-test (*P < 0.05).

Enzymatic activity	Col-0	L1	L2	L3
Illuminated (EoD)				
NADH-HPR	1130 ± 81	1175 ± 46	1295 ± 63	1269 ± 75
NADPH-HPR	157 ± 21	205 ± 37	167 ± 23	172 ± 18
NADH-GR	79 ± 12	73 ± 20	111 ± 33	90 ± 25
NADPH-GR	170 ± 37	161 ± 45	160 ± 40	133 ± 31
Darkness (EoN)				
NADH-HPR	1377 ± 69	1558 ± 138	1405 ± 136	1642 ± 172
NADPH-HPR	63 ± 10	123 ± 5*	126 ± 4*	121 ± 4*
NADH-GR	59 ± 7	66 ± 3	62 ± 5	55 ± 5
NADPH-GR	77 ± 14	77 ± 10	77 ± 18	68 ± 11

### Daytime serine depletion induces the phosphoserine pathway of serine synthesis

**Fig. 7.**
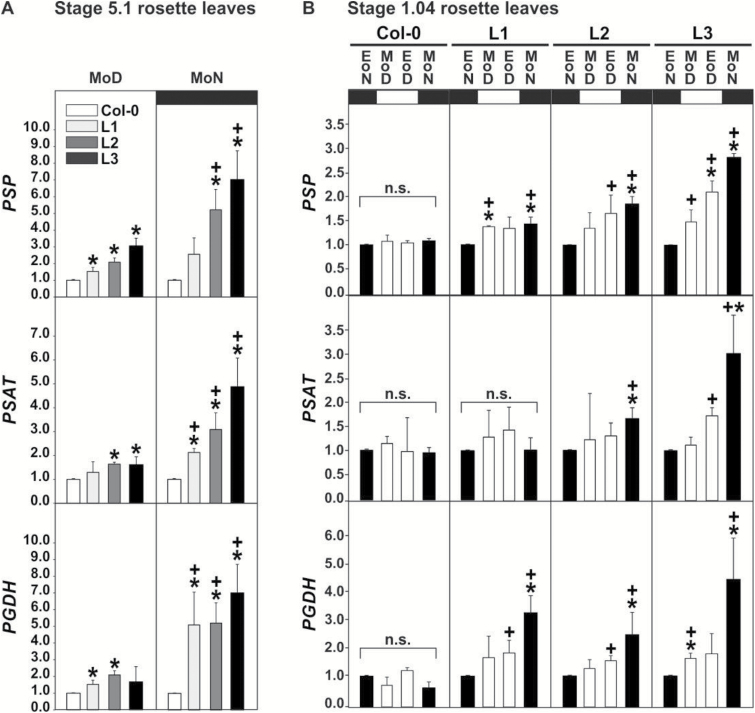
Transcript levels of genes encoding enzymes of the phosphoserine pathway. (A) Rosette leaves of mature plants grown in soil to stage 5.1 were harvested at the middle of the day (MoD) and the middle of the night (MoN). Fold values are mean±SD (*n*=3) relative to the respective wild type value, which was arbitrarily set to 1. Asterisks indicate significant changes compared with the wild type value. Pluses indicate significant changes compared with the corresponding MoD value of the same genotype. (B) Rosette leaves from six seedlings per genotype grown on MS to stage 1.04 were pooled at four time points during the day/night cycle (EoN, MoD, EoD, MoN). Fold values are mean±SD (*n*=3) relative to the individual mean EoN values, which were arbitrarily set to 1. Asterisks indicate significant changes compared with the corresponding wild type time point. Pluses indicate significant changes compared with the EoN value of the same genotype. Comparisons are based on Student’s *t*-test (*^,+^*P* < 0.05).

The phosphoserine pathway is considered the main route of serine synthesis in plants at night (Benstein *et al.*, 2013; Cascales-Miñana *et al.*, 2013; Ros *et al.*, 2014). Moreover, serine is a suggested signal for the transcriptional and/or metabolic regulation of several key processes in plant and mammalian cells (Ye *et al.*, 2012; Timm *et al.*, 2013; Häusler *et al.*, 2014). We therefore examined whether low daytime levels of serine in the *SGAT* overexpressors coincide with changes in the expression of genes encoding phosphoserine phosphatase (*PSP*), phosphoserine aminotransferase (*PSAT*) and 3PGA dehydrogenase (*3-PGDH*). This experiment was performed by using quantitative real-time PCR (qRT-PCR) with leaf RNA isolated from stage 5.1 plants at the middle of the day (MoD) and the middle of the night (MoN) and additionally with leaf RNA isolated from stage 1.04 seedlings at EoD, EoN, MoD and MoN (Fig. 7). The seedling experiment showed almost unchanged expression of all three phosphoserine pathway genes during the day–night cycle in the wild type, but a distinct daytime induction and an up to three-fold (*PSP*, *PSAT*) and four-fold (*3PGDH*) night-time induction of gene expression in the *SGAT* overexpressors. Higher transcription was associated with the gradually lower leaf serine contents of the transgenic lines (L1<L2<L3). All three genes were strongly upregulated during the day in the mature overexpressor plants, too, with an even higher induction at MoN (total increase five- to seven-fold in line L3) in combination with slight but significant increases already at MoD. In light of known developmental and sucrose effects on gene expression (for example Stitt *et al.*, 2002), the comparison of plants at developmental stages 1.04 and 5.1 was important for our experiments because it confirmed that transcription data obtained with MS-grown seedlings are a maybe rough but informative proxy for the gene expression in mature leaves.

### SGAT overexpression alters the diurnal transcriptional regulation of photorespiration

**Fig. 8.**
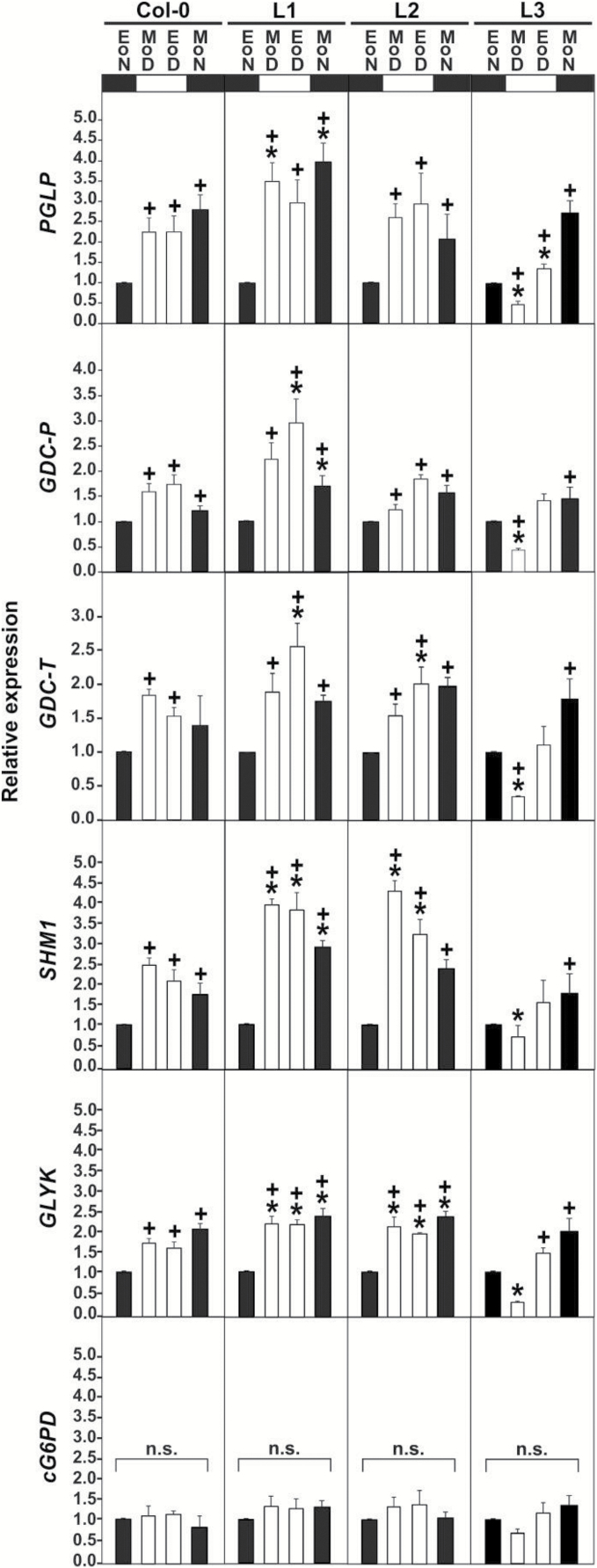
Expression of marker genes of the photorespiratory pathway. Wild type and *SGAT* overexpressors were grown on MS to stage 1.04. Cytosolic glucose-6-phosphate dehydrogenase (cG6PD) was used as a control. See Fig. 7B for further details.

In our previous study, serine was externally applied to Arabidopsis seedlings, in which its higher concentration abolished normal light induction of the expression of photorespiratory genes. We now used the *SGAT* overexpressor plants to study whether low-serine conditions would induce an opposite effect, namely a transcriptional induction of the genes encoding phosphoglycolate phosphatase (*PGLP*), GDC P-protein (*GDC-P*), GDC T-protein (*GDC-T*), mitochondrial serine hydroxymethyltransferase 1 (*SHM1*), and glycerate 3-kinase (*GLYK*). Considering the methodological point made in the previous paragraph, this experiment was performed with stage 1.04 rosette leaves harvested at EoN, MoD, EoD, and EoN (Fig. 8). Expression of cytosolic glucose-6-phosphate dehydrogenase (cG6PD) provided an internal control as it is basically unaltered over the diel cycle in all genotypes. In leaves of line L1 (about 50% daytime serine contents relative to the wild type), we observed significantly elevated transcript levels for all five genes compared with the wild type. In line L2 (about 30% daytime serine contents), this effect was distinctly visible only for *SHMT1* and *GLYK* but less pronounced for *PGLP*, *GDC-P* and *GDC-T*. In line 3 (about 20% daytime serine contents), gene expression was not stimulated at all during the day and was even lower than in the wild type. The changes in gene expression observed during the day became essentially normalized during the following night. Also at 1% CO_2_ (see Supplementary Fig. S2), relative expression of the five genes at EoD *vs.* EoN typically was not significantly different from the wild type in the *SGAT* overexpressors. Opposite to transcriptional induction by light in normal air, we interestingly observed that all five ‘photorespiratory’ genes were repressed at daytime under this non-photorespiratory condition in all genotypes. Daytime repression was significantly stronger in line L1 for *GDC-T*, *SHM1* and *GLYK*.

## Discussion

Earlier studies had shown SGAT is an essential component of the photorespiratory pathway (Somerville and Ogren, 1980) and also works as an aspartate aminotransferase (for example Murray *et al.*, 1987). It was also shown that less SGAT results in some accumulation of serine in barley and it was suggested that, overall, the enzyme exerts no control on photosynthesis (Wingler *et al.*, 1999*a*). In this work, we used an overexpression approach to more closely examine the role of SGAT for metabolic regulation particularly of the photorespiratory pathway and serine biosynthesis during the day and night. In light of the importance of both processes for growth, we also wanted to test whether elevated SGAT activity would change the plant’s overall performance.

In contrast to previous studies with other photorespiratory enzymes, elevated leaf SGAT activity did not improve but decreased photosynthetic performance and resulted in an altered phenotype characterized by smaller leaf numbers and reduced leaf expansion (Table 1 and Fig. 3). The smaller fresh weight in combination with unchanged dry weight, although the ratio of these parameters is significantly different from the wild type only in line L3, indicates leaves contain less water than the wild type.

The stronger response of photosynthesis to increasing O_2_ concentrations visible particularly in the values for Γ may indicate a higher extent of out-of-pathway decarboxylation of photorespiratory metabolites as it also occurs as the result of other interventions into photorespiration. The mechanism by which this could occur is not exactly known; some authors discussed non-enzymatic decarboxylation of glyoxylate and possibly hydroxypyruvate as potential routes (for example Walton and Butt, 1981; Cousins *et al.*, 2011). Research in recent years has shown that photorespiration is not a closed system but a branched regulated network with multiple input and output nodes (Florian *et al.*, 2013; Ma *et al.*, 2014; Obata *et al.*, 2016), and it is equally possible that the additional CO_2_ is produced for instance by pyruvate decarboxylase (Mithran *et al.*, 2014), which is known to accept glyoxylate as a substrate (Davies, 1967), or in pathways such as the TCA cycle, the photorespiratory folate cycle and/or the oxidative pentose phosphate pathway. Whilst the very considerably reduced daytime contents of serine and to a lesser extent asparagine are remarkable metabolic features of all *SGAT* overexpressing transgenic lines, higher SGAT activity had only little further impact on the leaf metabolomes in general, at day and night (Figs 5 and 6, and Supplementary Table S5). The most interesting other changes are related to the night-time operation of the TCA cycle (reduced 2OG in all lines) and the associated GABA shunt (reduced GABA in two lines). One possible explanation is that more 2OG could be needed to provide sufficient glutamate as the ultimate amino donor for nocturnal serine synthesis and that this, because 2OG is also the precursor for GABA synthesis, also leads to the reduced GABA levels. Overall, these data suggest that *SGAT* overexpression rather specifically alters carbon and nitrogen flow within and into the photorespiratory pathway and, as discussed in the paragraph after next, non-photorespiratory pathways of serine biosynthesis.

Concerning serine, our results match studies with heterozygous barley mutants, which showed about doubled serine contents with no significant change of photosynthetic CO_2_ fixation at 50% SGAT activity (Wingler *et al.*, 1999*a*,b). Likewise, they also correspond to more recent experiments with transgenic rice, in which a five-fold higher or about 90% lower serine content relative to the wild type was observed in plants with about 14% or 250%, respectively, leaf SGAT activity (Zhang *et al.*, 2015). Mechanistically, the impact of a varying SGAT activity on leaf serine levels in the daytime can be best explained by the deceleration or acceleration of the photorespiratory conversion of serine and glyoxylate to hydroxypyruvate and glycine as shown in Fig. 1. Our result that hydroxypyruvate contents at EoD were not consistently altered in the transgenic lines (lower only in L1; Fig. 6A and Supplementary Table S5) may reflect the high buffering capacity of the peroxisomal HPR1 plus cytosolic HPR2 reduction system in photorespiration (Timm *et al.*, 2011) and, in combination with the unaltered glycerate levels, suggests that GLYK has only little control over the photorespiratory pathway.

The daytime serine depletion in the *SGAT* overexpressors becomes fully normalized during the following night, and this is accompanied by a drop in EoN hydroxypyruvate levels (Fig. 5 and Supplementary Table S5). From the perspective of day–night metabolic regulation, this is a remarkable effect because it indicates activation of nocturnal serine biosynthesis. Plants produce serine via three routes: the photorespiratory pathway (the major route in photosynthesizing cells), the phosphoserine pathway (the major route at night and in heterotrophic tissue), and possibly the glycerate pathway (Ros *et al.*, 2014). Formally, the glycerate pathway is the reverse sequence of the photorespiratory pathway from 3-phosphoglycerate to serine, comprising 3PGA phosphatase, D-glycerate dehydrogenase, and SGAT (Kleczkowski and Givan, 1988). Operation of this pathway is still hypothetical mainly because a 3PGA phosphatase is not known in plants. The respective enzymatic activity was demonstrated in leaf extracts (Randall *et al.*, 1971) but is reportedly due to an unspecific acid phosphatase (Robinson, 1982). For the oxidation of glycerate to hydroxypyruvate, the cytosolic NAD(P)H-dependent HPR2 showed significantly enhanced nocturnal activity in the transgenic lines (Table 2) but is not a very likely candidate enzyme because it executes an essentially irreversible reaction (Kleczkowski and Randall, 1988). By contrast, the peroxisomal enzyme HPR1 catalyses a reversible reaction (Kohn and Warren, 1970) and displays a high nocturnal activity in the wild type, much higher than HPR2, plus some additional though not statistically significant induction in the transgenic lines (Table 2). The hydroxypyruvate produced would then be processed to serine by SGAT, likely using the amino donors asparagine or alanine (reactions SGAT^9.4^ and SGAT^6.9^, grey box in Fig. 1). The asparagine-dependent SGAT reactions with pyruvate (which potentially allows regeneration of alanine) and hydroxypyruvate actually show the highest *V*_max_*in vitro*, up to ten-fold higher than the *V*_max_ of the eponymous serine:glyoxylate transamination (Zhang *et al.*, 2013). Indeed, under the non-photorespiratory condition of 1% CO_2_, a loss-of-function mutant of HPR1 accumulates 50–70% less serine than the wild-type at night (Fig. 7 in Timm *et al.*, 2012*b*). After exposing the high-CO_2_-grown plants to normal air, the observed pattern was less clear due to serine accumulation following onset of photorespiration. Summarizing, whilst we presently assume that the SGAT-related drop in the EoN content of hydroxypyruvate in all transgenic lines (significant in two lines, Fig. 6B) suggests night-time operation of the glycerate pathway in leaves of Arabidopsis, such a conclusion would require more direct evidence.

On the other hand, sound evidence has established the phosphoserine pathway as the major source of serine during the night and in non-photosynthesizing organs (Ho and Saito, 2001; Benstein *et al.*, 2013; Cascales-Miñana *et al.*, 2013; Ros *et al.*, 2014). Seedlings and mature plants of the *SGAT* overexpressors consistently displayed higher expression of the genes encoding *3PGDH, PSAT* and *PSP* during the day and even more at night (Fig. 7). The extent of induction correlates with SGAT activity and serine depletion. This coincidence of altered SGAT activity, changes in gene expression and metabolic effects strongly suggests that daytime serine depletion activates the phosphoserine pathway at night and to some extent already during the day, resulting in a normalization of leaf serine contents under the condition of low to zero photorespiratory flux.

Following up from the observed down-regulation of marker genes of photorespiration after external serine application to Arabidopsis leaves (Timm *et al.*, 2013), we finally wanted to know whether these genes would be transcriptionally up-regulated in the low-serine *SGAT* overexpressor plants. This experiment was performed with seedlings to ensure compatibility with our previous study and also because the data in Fig. 7 had shown that transcription data obtained with seedling leaves are a suitable proxy for gene expression in mature leaves. The qRT-PCR data indeed show significant transcriptional upregulation of all five examined genes, *PGLP*, *GDC-P*, *GDC-T*, *SHM1* and *GLYK*, in line L1 relative to the wild type, particularly at EoD and MoN (Fig. 8). The upregulation pattern was less uniform in line L2, and we observed even lower daily expression of all examined genes in line L3, particularly at MoD. The night values remained essentially unaltered in comparison with the wild type. The same pattern of a distinctly elevated daytime gene expression in line L1, an irregular response in line L2, and distinctly lower expression in line L3 was consistently found in several repeats of this experiment. These data demonstrate that enhanced SGAT activity alters expression of several marker genes of photorespiration, likely via a route that involves serine as a metabolic signal as we have suggested earlier.

By contrast to the phosphoserine pathway, photorespiration not only produces but simultaneously consumes serine. Based on this, our current interpretation of the observed dial gene expression patterns is as follows. First, daytime induction of the phosphoserine pathway is highest in lines L2 and L3, which exhibit the strongest serine deprivation (Figs 5 and 7). This is intuitively to be expected because serine synthesis counteracts serine deprivation. Second, elevated serine levels prevent the otherwise typical transcriptional light induction of at least some key ‘photorespiratory’ genes (Timm *et al.*, 2013). In line with this previous report, the reciprocal experiment shown in Fig. 8 suggests that moderately lower daytime serine contents (in line L1 and to some extent L2) induce expression of these genes. When daytime leaf serine contents, however, become very low (as in line L3), signalling severe serine deprivation of cellular metabolism, the induction effect inverts into a clear MoD repression of all five examined genes of the photorespiratory pathway. We assume that this could serve downregulation of the photorespiratory pathway, reducing its capacity to consume serine for glycerate production; however, this is purely speculative at present. Quantitatively, the EoD serine contents of line L3 was 20% to 50% lower than in line L2 (HPLC data in Fig. 5*vs*. GC-MS data in Fig. 6A), which is not a minor difference. Hence, it is also possible that the physiological range for serine to properly act as a metabolic signal could be restricted, with a lower concentration threshold at about L1 leaf serine level (50% of wild-type level) that is undershot in lines L2 (30% of wild-type level) and even more in L3.

The lower daytime contents of asparagine in the *SGAT* overexpressor lines support the hypothesis by Ta *et al.* (1985) and Ta and Joy (1986) that asparagine in addition to glutamate is involved into the photorespiratory conversion of glyoxylate to glycine. It is interesting to note that the steady-state asparagine-to-aspartate ratio is low in the light and high in the dark in leaves of Arabidopsis, which is due to asparagine accumulation (Lea *et al.*, 2007). Thus, import of extra-peroxisomal asparagine amino nitrogen into photorespiration may become more relevant when photorespiration increases (for example in the morning) or becomes temporarily higher during the day. A future quantification of the dynamics of daytime fluxes through the transaminase network in leaves is clearly desirable. It already appears that excess SGAT activity in combination with more import of asparagine nitrogen into photorespiration likely would compete with nitrogen export from the leaf. We hence speculate that SGAT activity must be well controlled to ensure adequate photorespiratory carbon flux and, at the same time, avoid negative effects on the whole-plant nitrogen balance.

## Supplementary Material

Supplementary Data

## Abbreviations:

2OG: 2-oxoglutarate
2PG: 2-phosphoglycolate
2OS: 2-oxosuccinamate
3PGA: 3-phosphoglycerate
GDC: glycine decarboxylase
HPR: hydroxypyruvate reductase
SGAT: serine:glyoxylate aminotransferase
SHMT: serine hydroxymethyltransferase.
