# Supplementary material for: High serine:glyoxylate aminotransferase activity lowers leaf daytime serine levels, inducing the phosphoserine pathway in Arabidopsis
Source: J Exp Bot. 2016 Dec 23;68(3):643–56. doi: 10.1093/jxb/erw467 (PMC5441925; doi:10.1093/jxb/erw467)
Supplement: Supplementary Data [file erw467_Supplementary_Data.zip › supplementary_figures_S1_S2_tables_S1_S5.pdf]

Supplementary data

Supplementary Figure S1. Generation of *SGAT* overexpressors and genotyping.

(A) Outline of the *FpSGAT* overexpression construct. **(B)** PCR detection of the overexpression construct showing genomic integration of the 1206 bp full-length *FpSGAT* (B1) fused to the *STLS1* promotor (B2) and the S16 loading control (B3). (**C)** RT-PCR verification *FpSGAT* expression (C1) and the S16 calibration control (C2).

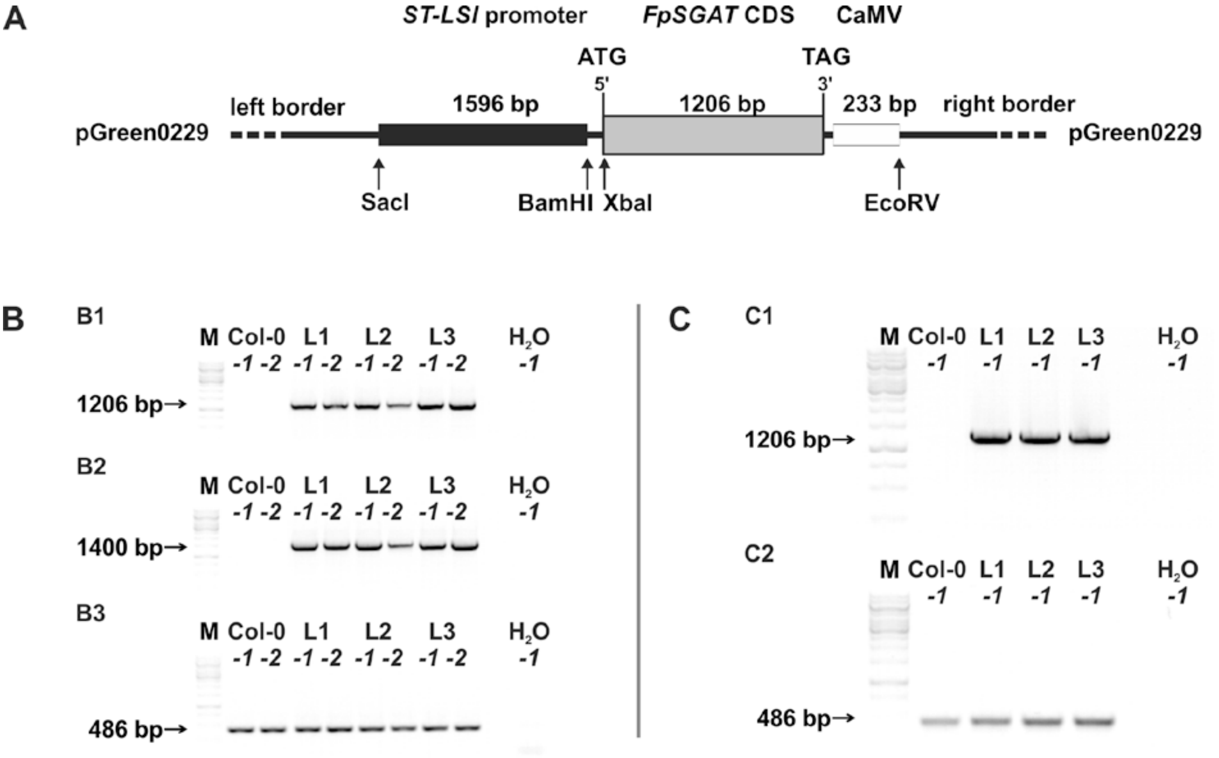

Supplementary Figure S2. Day/night expression of marker genes of the photorespiratory pathway at 1% CO_2.

Wild type and *SGAT* overexpressor plants were grown on solidified MS medium to stage 1.04. Rosette leaves from 6 individual plants per genotype were pooled at two time points during the day/night cycle (EoN - end of night, EoD - end of day). The 'fold' values are mean ± SD (N = 3) relative to the corresponding wild-type EoN value. Asterisks indicate significant changes compared to the wild type based on Student's t-test (*p<0.05; n.s., not significant).

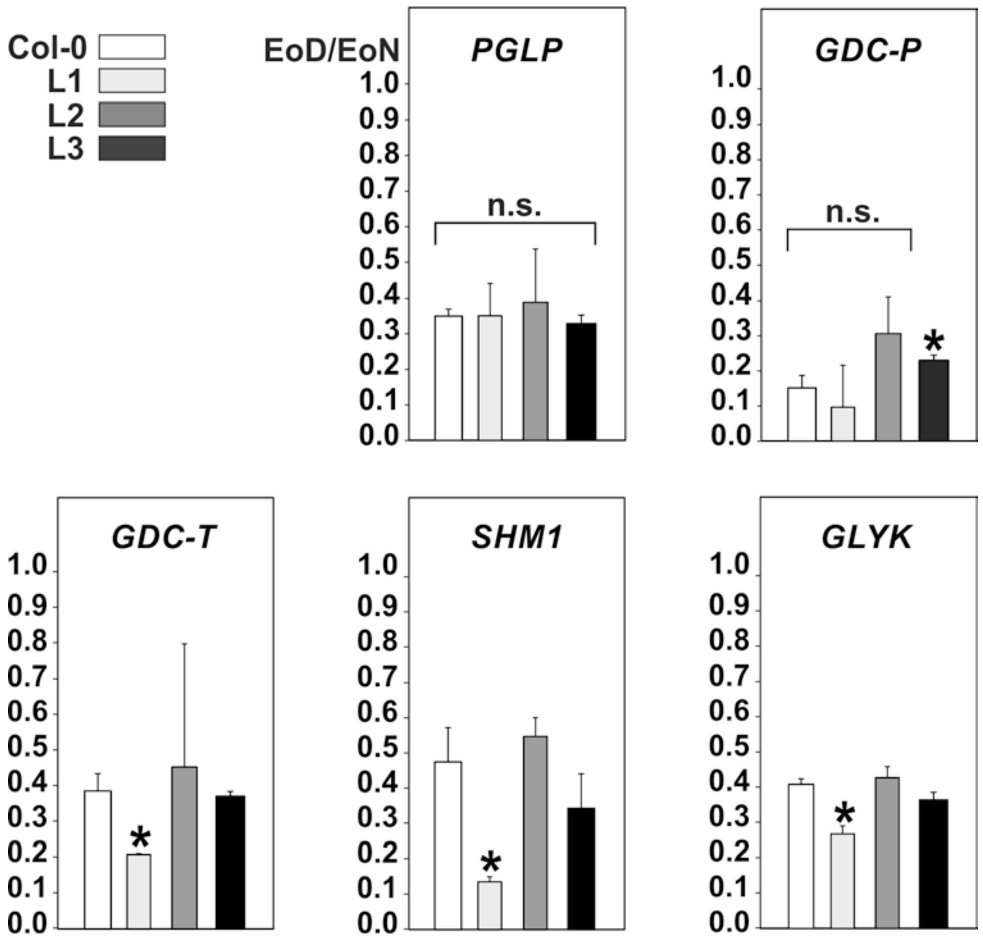

Supplementary Table S1. List and sequences of primers used in this research.

Primer	Sequence (5' to 3')	Fragment size (bp)
FpSGAT-cDS-fw	GGA TCC ATG GAC TAC ACT TAT GCA CCA GGA	1206
FpSGAT-cDS-rev	CCC GGG TCA AAT CCT TGA TGG GAT CAT GGG	
ST_LS1-fw-1200	AAC AAC CCA TAG AGG AAC CAA TCA	1400
FpSGAT-cDS-rev	CCC GGG TCA AAT CCT TGA TGG GAT CAT GGG	
S16-fw-RT	GGCGACACAACCAGCTACTGA	432
S16-rev-RT	CGGTAACTCTTCTGGTAACGA	
PGLP-fw-RT	CAG AAT GGC GGT TGT AAG AC	328
PGLP-rev-RT	GGC TCC CTA ATT TGC TAT GC	
GDCP-fw-RT	TAT GTC CAA TGC GTC GCT TC	327
GDCP-rev-RT	AGC AAA TCC GTA GCC ATC AC	
GDCT-fw-RT	GCA ATC AAT AAC CCG TCG TC	363
GDCT-rev-RT	TCA ATG GCA CCT CCT TTC TC	
SHM1-fw-RT	GCC CAG TGA AGC TGT TGA TG	365
SHM1-rev-RT	AGT TGG CAG GAG ATC CAG AC	
GLYK-fw-RT	ATG GCC TGA AGT TGA AGG AC	336
GLYK-rev-RT	TCC GCG TAA AGA GTT GGA AG	
PSP-fw-RT	GCG GTA TGT TTC GAT GTG GAC	381
PSP-rev-RT	CTC GCC GGA GTT TCC AAA TAG	
PSAT-fw-RT	AAC TGG CGT GGA TCT GGT ATG	351
PSAT-rev-RT	ATA CTT GGC GTC CGA ACT CTG	
3-PDGH-fw-RT	GCT TTC CTT CAC CGC CGT TAC	333
3-PGDH-rev-RT	CTT CGC CGC CTC AAA CAC TTC	
cG6PD-fw-RT	TGT GGT TCC GAT AAG CGA TGA C	373
cG6PD-rev-RT	ATA CCG TTG CCC ATA CGA CAA G	

Supplementary Table S2. Leaf amino acids contents at stage 5.1 at the end of the day.

Rosette leaf samples were harvested after 9 h illumination and steady-state contents of amino acids analysed by HPLC. Values are means ± SD of four independent biological replicates. Values in bold were significantly different from the wild type based on Student's t-test (*p < 0.05). Amino acids marked with an asterisk were used for Figure 5.

**Leaf amino acid contents in SGAT lines compared to the wild type**				
µmol g^{-1} FW	**Col-0**	**L1**	**L2**	**L3**
Alanine*	4.09 ± 0.35	**2.73 ± 0.26**	**3.46 ± 0.31**	3.96 ± 0.39
Arginine	0.22 ± 0.07	0.27 ± 0.07	0.17 ± 0.03	0.17 ± 0.03
Asparagine*	2.02 ± 0.20	1.74 ± 0.30	**1.16 ± 0.18**	**1.21 ± 0.13**
Aspartate	4.71 ± 1.00	4.77 ± 0.52	4.84 ± 1.05	5.73 ± 1.45
Glutamate	7.52 ± 1.37	**11.0 ± 1.86**	**13.4 ± 2.53**	**14.5 ± 2.05**
Glutamine	27.37 ± 2.53	31.4 ± 4.46	32.1 ± 8.36	**34.6 ± 4.59**
Glycine	2.89 ± 1.09	2.02 ± 0.92	2.22 ± 1.20	2.64 ± 0.67
Histidine	0.19 ± 0.07	0.14 ± 0.03	0.15 ± 0.07	0.22 ± 0.18
Isoleucine	0.23 ± 0.07	0.21 ± 0.03	0.20 ± 0.05	0.26 ± 0.06
Leucine	0.21 ± 0.05	0.20 ± 0.04	0.22 ± 0.05	0.28 ± 0.10
Lysine	0.09 ± 0.03	0.07 ± 0.01	0.08 ± 0.04	0.14 ± 0.11
Methionine	0.04 ± 0.01	0.05 ± 0.01	0.03 ± 0.01	0.03 ± 0.01
Phenylalanine	0.21 ± 0.05	0.23 ± 0.03	0.23 ± 0.04	0.30 ± 0.05
Serine*	11.33 ± 1.17	**5.48 ± 0.36**	**3.18 ± 0.53**	**2.49 ± 0.57**
Threonine	5.38 ± 0.76	5.32 ± 0.43	5.40 ± 0.85	6.08 ± 0.97
Tyrosine	0.21 ± 0.09	0.23 ± 0.07	0.26 ± 0.09	0.52 ± 0.47
Tryptophan	0.13 ± 0.04	0.09 ± 0.04	0.13 ± 0.08	0.18 ± 0.05
Valine	1.64 ± 0.56	1.71 ± 0.46	1.71 ± 0.53	2.12 ± 0.94
Gly/Ser ratio	0.23 ± 0.08	0.32 ± 0.14	**0.67 ± 0.27**	**1.00 ± 0.33**
Total*	67.44 ± 8.12	63.66 ± 10.9	70.60 ± 13.7	78.95 ± 10.6

Supplementary Table S3. Leaf amino acids contents at stage 5.1 at the end of the day of plants grown in air with 1% CO_2.

Rosette leaf samples were harvested after 9 h illumination and steady-state contents of amino acids analysed by HPLC. Values are means ± SD of four independent biological replicates. Values in bold were significantly different from the wild type based on Student's *t*-test (*p < 0.05).

Leaf amino acid contents in SGAT lines compared to the wild type				
µmol g^{-1} FW	**Col-0**	**L1**	**L2**	**L3**
Alanine	9.96 ± 2.41	9.57 ± 2.03	8.91 ± 3.66	10.45 ± 4.36
Arginine	0.39 ± 0.18	0.28 ± 0.02	0.37 ± 0.13	0.91 ± 0.49
Asparagine	4.60 ± 0.68	3.66 ± 0.61	3.23 ± 1.10	4.77 ± 1.84
Aspartate	4.09 ± 0.58	3.22 ± 0.85	4.97 ± 1.33	4.03 ± 0.35
Glutamate	10.2 ± 1.14	10.6 ± 3.63	11.9 ± 3.82	11.9 ± 3.10
Glutamine	31.1 ± 3.69	26.7 ± 5.50	26.4 ± 5.95	22.6 ± 4.79
Glycine	1.40 ± 0.54	1.10 ± 0.52	1.51 ± 0.53	2.10 ± 1.24
Histidine	0.26 ± 0.15	0.13 ± 0.01	0.22 ± 0.11	**0.72 ± 0.16**
Isoleucine	0.54 ± 0.26	0.28 ± 0.01	0.32 ± 0.15	1.21 ± 0.37
Leucine	0.67 ± 0.38	0.29 ± 0.03	0.36 ± 0.28	**1.59 ± 0.21**
Lysine	0.32 ± 0.15	0.19 ± 0.01	0.19 ± 0.08	0.65 ± 0.31
Methionine	0.013 ± 0.017	**0.002 ± 0.001**	**0.004 ± 0.002**	**0.004 ± 0.002**
Phenylalanine	0.52 ± 0.23	0.35 ± 0.03	0.29 ± 0.08	0.90 ± 0.22
Serine	6.49 ± 2.01	6.44 ± 0.96	4.14 ± 0.99	5.44 ± 1.61
Threonine	8.74 ± 1.65	7.38 ± 1.52	6.41 ± 2.51	7.79 ± 2.20
Tyrosine	1.00 ± 0.49	0.51 ± 0.05	0.74 ± 0.33	2.72 ± 1.30
Tryptophan	0.42 ± 0.09	0.20 ± 0.12	0.25 ± 0.18	0.48 ± 0.06
Valine	1.18 ± 0.54	0.84 ± 0.02	1.10 ± 0.51	**2.26 ± 0.33**
Gly/Ser ratio	0.23 ± 0.04	0.18 ± 0.11	**0.46 ± 0.04**	0.36 ± 0.15
Total	84.6 ± 7.52	69.7 ± 13.0	71.2 ± 8.12	76.6 ± 10.1

Supplementary Table S4. Leaf amino acids contents at stage 5.1 at the end of the night.

Rosette leaf samples were harvested after 13 h darkness and steady-state contents of amino acids analyzed by HPLC. Values are means ± SD of four independent biological replicates. Values in bold were significantly different from the wild type based on Student's *t*-test (*$p < 0.05$). Amino acids marked with an asterisk were used in Figure 5.

**Leaf amino acid contents in SGAT lines compared to the wild type**				
µmol g^{-1} FW	**Col-0**	**L1**	**L2**	**L3**
Alanine*	3.42 ± 0.60	3.68 ± 0.87	3.29 ± 0.84	3.36 ± 0.46
Arginine	0.09 ± 0.03	0.10 ± 0.03	0.09 ± 0.03	0.11 ± 0.06
Asparagine*	1.48 ± 0.27	1.33 ± 0.16	1.33 ± 0.16	1.62 ± 0.21
Aspartate	4.46 ± 1.30	5.27 ± 1.41	5.06 ± 0.81	4.99 ± 0.58
Glutamate	11.06 ± 2.26	11.71 ± 0.59	11.24 ± 1.16	12.07 ± 1.64
Glutamine	8.38 ± 0.79	7.65 ± 0.86	7.65 ± 0.86	7.97 ± 3.60
Glycine	3.00 ± 0.78	1.92 ± 0.38	**0.41 ± 0.16**	**0.33 ± 0.17**
Histidine	0.40 ± 0.25	0.40 ± 0.14	0.27 ± 0.19	0.23 ± 0.15
Isoleucine	0.31 ± 0.21	0.35 ± 0.10	0.26 ± 0.13	0.30 ± 0.13
Leucine	0.40 ± 0.26	0.33 ± 0.12	0.16 ± 0.09	0.31 ± 0.30
Lysine	0.21 ± 0.13	0.26 ± 0.07	0.21 ± 0.09	0.23 ± 0.10
Methionine	0.013 ± 0.006	0.009 ± 0.001	**0.005 ± 0.001**	**0.003 ± 0.001**
Phenylalanine	0.32 ± 0.15	0.41 ± 0.06	0.34 ± 0.12	0.38 ± 0.05
Serine*	4.93 ± 1.13	5.48 ± 0.36	4.30 ± 1.95	4.45 ± 1.66
Threonine	4.10 ± 0.62	5.27 ± 2.02	3.55 ± 0.70	4.38 ± 0.18
Tyrosine	0.72 ± 0.49	0.79 ± 0.30	0.47 ± 0.32	0.54 ± 0.32
Tryptophan	0.32 ± 0.09	0.38 ± 0.12	0.27 ± 0.08	0.28 ± 0.06
Valine	0.80 ± 0.37	0.83 ± 0.21	0.60 ± 0.23	0.67 ± 0.29
Gly/Ser ratio	0.73 ± 0.12	**0.46 ± 0.15**	**0.14 ± 0.06**	**0.08 ± 0.02**
Total*	49.71 ± 10.2	51.43 ± 5.89	42.87 ± 6.80	44.8 ± 5.49

Relative metabolite contents at the end of the day (EoD) in *SGAT* overexpressors grown in normal air.

Significantly reduced compared to the wild type ($p < 0.05$)
Significantly elevated compared to the wild type ($p < 0.05$)

Compound	EoD_WT rv to WT	EoD_WT rse to WT	EoD_L1 t-test to WT	EoD_L1 rv to WT	EoD_L1 rse to WT	EoD_L2 t-test to WT	EoD_L2 rv to WT	EoD_L2 rse to WT	EoD_L3 t-test to WT	EoD_L3 rv to WT	EoD_L3 rse to WT
2-Methyl-malate	1	0,1515198	0,8732189	1,0260922	0,0460519	0,4532943	0,8084737	0,1899614	0,2195743	1,23429	0,0893466
2-Oxo-glutarate	1	0,1466243	0,325228	0,7736527	0,1486055	0,8482247	0,9545686	0,1769718	0,1327571	1,5335375	0,2830896
Alanine	1	0,1125905	0,0302003	0,4475114	0,1376229	0,5171358	1,2555709	0,359971	0,1278783	0,748922	0,0958116
Alanine, beta	1	0,0855562	0,0286161	0,6437242	0,1027734	0,8683368	1,0265303	0,1292357	0,8660319	1,02922	0,1442728
Argenine	1	0,1810584	0,1204158	0,6472176	0,0917662	0,2810466	0,7704516	0,0815502	0,1259337	0,6485123	0,0977002
Ascorbate	1	0,054987	0,2163185	1,570131	0,4211425	0,1311172	1,6589699	0,3879531	0,0203064	1,9939553	0,2319212
Asparagine	1	0,1969294	0,0425074	0,4451078	0,1192953	0,0396322	0,4821383	0,075621	0,0175272	0,3589537	0,0860758
Aspartate	1	0,1218069	0,2192134	0,7331064	0,1588886	0,569034	1,1329878	0,1879277	0,1048236	1,4354255	0,204571
Benzoate	1	0,1316884	0,4773318	1,2114309	0,2512068	0,760886	0,9533806	0,0676307	0,5138344	0,9019544	0,0570874
Citrate	1	0,2977227	0,0664936	0,260238	0,1006733	0,5775846	0,7871984	0,2139232	0,2958438	1,4955233	0,328116
Dehydroascorbate	1	0,2790021	0,3765954	0,6749235	0,2005912	0,1837271	1,7229876	0,4111328	0,1875756	1,8693997	0,5349816
Erythritol	1	0,1315563	0,2390331	0,8105778	0,0697294	0,190231	0,7942364	0,0579682	0,8022721	1,0403083	0,0832958
Ethanolamine	1	0,161636	0,3649869	0,7823238	0,1588923	0,1802898	0,6853225	0,1407951	0,2886604	0,7929262	0,0841092
Fructose	1	0,2221689	0,8612952	0,9359558	0,2768043	0,6040256	1,1679067	0,2176977	0,2140191	0,6302222	0,1602697
Fructose-6-phosphate	1	0,2162533	0,0564788	0,3235095	0,1982674	0,4582736	0,8141473	0,0490196	0,146045	1,6209584	0,3025975
Fucose	1	0,1311134	0,9501568	1,0134036	0,1612365	0,4053723	1,1524902	0,1138032	0,4531864	0,8696286	0,1007645
Fumarate	1	0,0529197	0,4461498	0,9440463	0,0455697	0,5463051	0,9554621	0,0468867	0,616808	0,9599509	0,0558548
Gaba	1	0,1951718	0,1163968	0,6210751	0,09081	0,1816814	1,5551658	0,3255069	0,4341771	0,8152815	0,1106508
Galactinol	1	0,2102975	0,8398239	1,0612436	0,2044705	0,5340015	1,1828245	0,1868794	0,2442352	0,6812363	0,1417416
Gentibiose	1	0,2235662	0,1008984	4,8261092	1,6411372	0,0519675	3,8049077	0,9210242	0,9399882	0,9461244	0,6421345
Glucose	1	0,1266321	0,8774072	0,9304335	0,4082329	0,4816295	0,8096789	0,2204641	0,0134231	0,427821	0,1150637
Glucose, 1,6-anhydro, beta-	1	0,1008381	0,2743651	0,7677407	0,1703152	0,5350742	0,9261211	0,0531606	0,5326342	0,895374	0,1248036
Glucose-6-phosphate	1	0,219548	0,2498186	0,6519112	0,1690316	0,5593371	0,8543114	0,0772768	0,0968879	2,0811204	0,4625122
Glutamate	1	0,0767277	0,1283996	0,7224113	0,1446145	0,9431862	0,9919484	0,0781148	0,7679194	1,0352609	0,0863185
Glutamine	1	0,1078017	0,7990078	1,0592247	0,197461	0,4944226	1,1377672	0,1594139	0,8712786	1,0218125	0,0733189
Glycerate	1	0,1592195	0,8479047	0,961675	0,1098882	0,3484236	0,83068	0,0595808	0,5251992	0,8829124	0,0756232
Glycerol	1	0,0693868	0,2660996	1,1480546	0,1025716	0,3857239	1,0842673	0,0601794	0,9051729	1,0127987	0,0775891
Glycerol-3-phosphate	1	0,2795012	0,1693946	0,5277455	0,1401638	0,7258265	0,8668522	0,2371332	0,812239	1,0992013	0,2917855
Glycine	1	0,1793764	0,3925686	0,7659722	0,1867945	0,3025118	0,7415734	0,151058	0,4703248	0,8386029	0,1148476
Glycolate	1	0,1032749	0,1822819	0,8373773	0,0416233	0,1313877	0,7963135	0,0634567	0,0383049	0,7054043	0,0590085
Hydroxyproline	1	0,48371	0,3664925	0,4830832	0,0972572	0,2100578	0,3075603	0,0971997	0,2478146	0,351786	0,1499841
Hydroxypyruvate	1	0,0996095	0,0237476	0,5767734	0,1059762	0,8528748	1,0467005	0,2225402	0,3207505	0,8628849	0,0828173
Inositol	1	0,0361677	0,2044347	0,8747177	0,0831505	0,4809734	0,9614019	0,0376727	0,3688386	0,9421363	0,0488275
Isoleucine	1	0,1730064	0,0187729	0,4824781	0,0331836	0,6293728	1,1406611	0,2206052	0,0340033	0,4637079	0,0460916
Lysine	1	0,1618589	0,0215578	0,5184422	0,0489849	0,3394937	1,4150369	0,3751888	0,7427989	0,9130147	0,1983812
Malate	1	0,0615027	0,0592618	0,802437	0,0655976	0,5673869	0,9440162	0,0709116	0,8559181	0,9826331	0,0692407
Maltose	1	0,1962064	0,0881803	1,5036582	0,1697639	0,063333	2,1157239	0,4792573	0,0031582	**2,5801763**	**0,3250811**
Melibiose	1	0,3087874	0,1791811	2,1082683	0,6865506	0,1799331	1,7996449	0,4481326	0,9258467	0,9595561	0,2862984
Methionine	1	0,2772324	0,395742	0,6759496	0,2219489	0,3034487	0,6456489	0,1177389	0,5269505	0,7771352	0,1724452
Nicotinate	1	0,1059513	0,6407783	0,9164337	0,1359423	0,3237514	1,1277116	0,0593805	0,7519985	0,9563209	0,0812863
Ornithine	1	0,1071836	0,0257945	0,6984461	0,026485	0,6863491	1,0705584	0,1299629	0,8217529	1,0567896	0,2191181
Phenylalanine	1	0,1398108	0,0472539	0,6139578	0,0872866	0,8207562	1,0454281	0,1345241	0,4993594	0,8572724	0,1454406
Picolinate	1	0,330638	0,1251937	0,4013947	0,1135293	0,2165404	0,506332	0,1049586	0,9506223	1,0291539	0,3144434
Proline	1	0,63159	0,607023	0,6283712	0,2498983	0,182993	0,0780403	0,0322811	0,3131083	0,2834625	0,2101699
Putrescine	1	0,1283539	0,4304207	0,7938726	0,2124825	0,2217864	1,4354623	0,3025691	0,915146	1,0164025	0,0759908
Pyruvate	1	0,105614	0,0204121	0,6673214	0,0464732	0,7476619	1,0758451	0,2017627	0,7895918	1,0349098	0,0696536
Raffinose	1	0,1869327	0,1983499	1,5432085	0,3272869	0,2826713	1,4305696	0,3151926	0,6429529	0,8412168	0,2685938
Rhamnose	1	0,1500371	0,7997849	0,9474392	0,1329293	0,583266	1,1176478	0,1408746	0,5881265	0,9036509	0,0816011
Salicylate	1	0,1454375	0,4302736	1,2896911	0,308361	0,1282279	2,0857294	0,5655243	0,0805605	1,6993532	0,2961141
Serine	1	0,1924055	0,0075711	**0,3097027**	**0,0301745**	0,0028978	**0,1752376**	**0,033097**	0,0015401	**0,0915288**	**0,0181959**
Shikimate	1	0,0303433	0,2033142	0,8052661	0,1372415	0,0742577	0,8904968	0,0438925	0,4075335	0,9092981	0,0992295
Sorbose	1	0,2479313	0,8226049	0,9090447	0,3044024	0,5252279	1,2425215	0,2680387	0,1704402	0,5557028	0,1598241
Spermidine	1	0,2074053	0,3048659	0,7370094	0,120558	0,5492328	0,8295341	0,1769678	0,1596911	0,6434065	0,0994866
Succinate	1	0,2110426	0,5392163	0,801225	0,2269621	0,1880917	0,6386582	0,1360584	0,6223406	0,8718317	0,1344389
Sucrose	1	0,1515603	0,2641425	0,7830903	0,0779049	0,8855537	0,9707848	0,1224226	0,3219577	0,7923727	0,1189528
Threonate	1	0,0619016	0,2676575	0,8534018	0,106348	0,580653	0,9352389	0,09393	0,5516027	0,91593	0,1202895
Threonine	1	0,1103984	0,0363136	0,6993057	0,0463929	0,037864	0,672201	0,0722785	0,0241175	0,6881688	0,0210103
Trehalose	1	0,2591086	0,7735729	0,8953078	0,2379037	0,9299518	0,9633517	0,309976	0,7155679	0,893157	0,1137387
Tryptophan	1	0,1954481	0,0498304	0,5148719	0,0772962	0,9279204	0,9742594	0,1945065	0,0829038	0,5755226	0,0877929
Tyrosine	1	0,1349798	0,0334216	0,5533658	0,1100753	0,3979439	1,4325137	0,4651723	0,2519759	0,7085324	0,193666
Valine	1	0,1402885	0,1053923	0,7154246	0,0680124	0,3782617	1,2007515	0,1632343	0,4871217	0,8215785	0,2007844

Abbreviations

EoD - End of day
rv - relative value
rse - relative standard error
WT - wild type
L1 - FpSGAT overexpressors line 1
L2 - FpSGAT overexpressors line 2
L3 - FpSGAT overexpressors line 3

Relative metabolite contents at the end of the night (EoN) in *SGAT* overexpressors grown in normal air.

Significantly reduced compared to the wild type ($p < 0.05$) (blue)
Significantly elevated compared to the wild type ($p < 0.05$) (red)

Compound	EoN_WT rv to WT	EoN_WT rse to WT	EoN_L1 t-test to WT	EoN_L1 rv to WT	EoN_L1 rse to WT	EoN_L2 t-test to WT	EoN_L2 rv to WT	EoN_L2 rse to WT	EoN_L3 t-test to WT	EoN_L3 rv to WT	EoN_L3 rse to WT
2-Methyl-malate	1	0,1417115	0,9701724	0,992067	0,1490112	0,7467584	1,1005196	0,2652128	0,8526813	1,0388262	0,1445614
2-Oxo-glutarate	1	0,2131075	0,0163029	0,1122366	0,1122366	0,0183003	0	0	0,0183003	0	0
Alanine	1	0,1622809	0,0348727	0,4756759	0,1279744	0,35084	0,7776247	0,155071	0,0215568	0,4486588	0,1055973
Alanine, beta	1	0,1865694	0,3494129	0,7480153	0,1716963	0,6264233	0,8579524	0,2096725	0,5005955	0,825062	0,1633988
Argenine	1	0,0814554	0,9363394	1,012655	0,1301606	0,5190713	1,0900726	0,1058473	0,804399	0,9513894	0,1659729
Ascorbate	1	0,2948823	0,0316181	0,2319034	0,0178569	0,0290709	0,2094972	0,0418961	0,023309	0,1704245	0,0319248
Asparagine	1	0,4191234	0,1929766	0,3878564	0,0989672	0,096152	0,2095002	0,0136827	0,1176388	0,2557006	0,0674174
Aspartate	1	0,2341628	0,3716491	0,7466164	0,1298048	0,2323946	0,6164178	0,1824766	0,4596498	0,7734561	0,1738657
Benzoate	1	0,0430823	0,0013247	**0,6361192**	**0,0620318**	0,0016458	**0,6488503**	**0,0620314**	0,0005887	**0,5761747**	**0,0642591**
Citrate	1	0,2790032	0,3861658	0,690767	0,1896937	0,1434351	0,4077029	0,235548	0,5910961	0,7791925	0,2753319
Dehydroascorbate	1	0,2847513	0,0089017	**0,0221074**	**0,0027669**	0,0086029	**0,0155072**	**0,0027341**	0,0260745	0,0179826	0,0046657
Erythritol	1	0,1423694	0,2887877	0,8032442	0,098602	0,5578061	0,8610181	0,1771377	0,7913336	0,9562535	0,0727951
Ethanolamine	1	0,172422	0,1723779	0,7114692	0,0856947	0,2995794	0,795362	0,0656431	0,7637954	1,0960675	0,2563733
Fructose	1	0,3315451	0,1319243	2,4594669	0,7687878	0,0299935	6,9875484	1,8391921	0,0336802	2,4800632	0,4495925
Fructose-6-phosphate	1	0,3351249	0,8392727	0,894672	0,3590293	0,2170896	0,3936755	0,2902464	0,7241819	0,8406694	0,2641478
Fucose	1	0,1137291	0,6959212	0,9143457	0,1781614	0,5776506	0,928016	0,0494985	0,1742786	0,7942378	0,0781567
Fumarate	1	0,0519149	0,2461013	0,8933013	0,061529	0,0334516	0,8600529	0,0168698	0,0634641	0,8793444	0,0210949
Gaba	1	0,1177784	0,0477298	0,6472191	0,0945492	0,3046998	0,776025	0,1668517	0,0101858	0,5900686	0,0341312
Galactinol	1	0,1152499	0,7921813	0,9159167	0,2756462	0,0455515	1,9872958	0,4011098	0,4850918	1,1680766	0,1986155
Gentibiose	1	0,729776	0,0476235	3,0397246	0,1582781	0,048488	4,3037688	1,2187819	0,0318087	3,9674281	0,7255124
Glucose	1	0,174548	0,3601109	3,416701	2,3370389	0,1383586	1,7273863	0,3821329	0,7261125	1,117175	0,2686634
Glucose, 1,6-anhydro, beta-	1	0,0559634	0,638879	0,9594879	0,061398	0,256568	0,8619038	0,0982018	0,1639477	0,8185663	0,1043322
Glucose-6-phosphate	1	0,3172772	0,2674085	0,5286097	0,2192126	0,1326832	0,3130888	0,2320656	0,3867663	0,639373	0,2072797
Glutamate	1	0,1368543	0,642257	0,9241111	0,0773999	0,1934733	0,7744765	0,0806599	0,3721923	0,8504401	0,0793996
Glutamine	1	0,2781479	0,1259242	0,4826783	0,119661	0,0472047	0,330585	0,0653687	0,0548808	0,3534188	0,0740616
Glycerate	1	0,1727333	0,3132407	1,3316402	0,2552135	0,171955	1,2653995	0,038217	0,3988834	1,1916776	0,1281891
Glycerol	1	0,1220586	0,0012149	**0,3685322**	**0,0424717**	0,0006281	**0,324839**	**0,0244595**	0,0009939	**0,3291644**	**0,0526703**
Glycerol-3-phosphate	1	0,2901715	0,6421805	0,8126104	0,2502628	0,7110056	0,8171338	0,3742981	0,7167979	1,1369929	0,2125299
Glycine	1	0,1003857	0,5785299	0,9231445	0,0868496	0,1769917	0,8274502	0,0592017	0,5629838	1,0812585	0,0897821
Glycolate	1	0,1308177	0,4192292	1,1697588	0,1504307	0,133371	1,2732864	0,0982386	0,8545064	1,0262416	0,0456717
Hydroxyproline	1	0,5661203	0,6373236	0,610871	0,5570475	0,7334796	0,7289926	0,5197747	0,2642245	0,2719674	0,2172097
Hydroxypyruvate	1	0,1456237	0,0118501	0,4263917	0,1005325	0,1203159	0,6713578	0,1205341	0,0065413	**0,3830072**	**0,0862913**
Inositol	1	0,044543	0,2508188	0,9196806	0,0471723	0,0223049	0,8376783	0,0362814	0,0113681	0,8525303	0,007113
Isoleucine	1	0,092734	0,4065699	0,7700072	0,2456306	0,2641736	1,5460275	0,4451636	0,9692945	1,0092824	0,2075053
Lysine	1	0,0704994	0,9230367	0,9830795	0,1543811	0,1064944	1,3782348	0,1956789	0,4874482	1,1363699	0,1644485
Malate	1	0,1417796	0,7436442	0,9415779	0,0983508	0,4975859	0,8707988	0,1138733	0,8652674	0,9736851	0,0495534
Maltose	1	0,1934267	0,8467916	0,9347334	0,2636899	0,1572311	0,6886403	0,0488826	0,1253277	0,6339394	0,0912445
Melibiose	1	0,3174658	0,5214627	1,3215	0,3594107	0,1067741	2,3304859	0,6599352	0,3142484	1,5352745	0,3843483
Methionine	1	0,1698915	0,5455709	0,8698976	0,1133878	0,6740668	0,8960655	0,1611824	0,1511783	0,6375461	0,1474631
Nicotinate	1	0,091242	0,7643556	1,0392894	0,0878635	0,7713582	1,0298037	0,0387671	0,3413539	1,146817	0,1128697
Ornithine	1	0,1780344	0,2191421	0,7525421	0,0524337	0,3167974	0,8048556	0,0413119	0,5163009	0,8349791	0,1630482
Phenylalanine	1	0,118014	0,0335397	0,5969163	0,104043	0,3356401	0,8324135	0,1133055	0,0581816	0,6561749	0,095628
Picolinate	1	0,2517205	0,1043037	0,4249879	0,1742325	0,2077543	0,4970739	0,2670265	0,3207289	0,6705431	0,1830534
Proline	1	0,8144237	0,2899223	0,0074754	0,002223	0,294571	0,0184917	0,0076831	0,2902528	0,0082656	0,0020615
Putrescine	1	0,2813389	0,0918805	0,438291	0,0832067	0,3122803	0,6476645	0,1660972	0,322908	0,6545483	0,1684693
Pyruvate	1	0,1621613	0,0098617	**0,4392715**	**0,0384035**	0,0546568	0,5980607	0,0751375	0,0825516	0,6129547	0,1084706
Raffinose	1	0,2313369	0,1870661	2,0632374	0,6996821	0,0229462	3,656443	0,9176262	0,1463251	1,8245627	0,457352
Rhamnose	1	0,0849132	0,2409226	0,8121282	0,1216148	0,4733707	0,9071906	0,0894742	0,0513161	0,7313115	0,0810189
Salicylate	1	0,1551021	0,9548324	1,0155114	0,2116252	0,1081709	2,4083299	0,7632119	0,1374435	1,4870562	0,2510438
Serine	1	0,1640696	0,6613136	0,9099237	0,1109095	0,6939936	1,1138189	0,2256421	0,870648	0,9576135	0,1913963
Shikimate	1	0,1492632	0,7975387	1,0752939	0,2414731	0,8963327	0,9733539	0,1302745	0,2687106	0,8001231	0,0774766
Sorbose	1	0,4060287	0,1408148	2,5985064	0,8505732	0,0318034	8,1166162	2,2271781	0,039805	2,6882996	0,5302321
Spermidine	1	0,1454169	0,175847	0,7255291	0,1140884	0,633365	0,891267	0,1641624	0,2734564	0,7653274	0,1335324
Succinate	1	0,1291493	0,5022931	1,2123967	0,2733726	0,5693746	1,1238647	0,1640351	0,1390429	1,2887587	0,1192278
Sucrose	1	0,1511544	0,549401	0,8709458	0,1406913	0,8102168	0,9613964	0,0365964	0,1406751	0,7091579	0,0937708
Threonate	1	0,0653974	0,6065724	1,0411083	0,0400808	0,7100881	1,0311635	0,0476066	0,5821837	1,03774	0,0075139
Threonine	1	0,1007144	0,5071817	0,8618091	0,1589174	0,9818091	1,007408	0,277358	0,2577642	0,7517112	0,1668075
Trehalose	1	0,2526341	0,9889769	1,0053593	0,2784832	0,1601142	0,5629372	0,1258954	0,0990888	0,5126049	0,0666059
Tryptophan	1	0,1989776	0,1785752	0,7012767	0,0380948	0,3419702	0,7878165	0,0672641	0,231041	0,7161169	0,0915026
Tyrosine	1	0,1062908	0,0434755	0,6120689	0,1143315	0,3085605	1,6037702	0,5450077	0,5923177	1,2342024	0,4062806
Valine	1	0,0946947	0,046909	0,6546554	0,1126328	0,6709031	1,1393608	0,3015058	0,2411168	0,7927886	0,1276678

Abbreviations

EoN - End of night
rv - relative value
rse - relative standard error
WT - wild type
L1 - FpSGAT overexpressors line 1
L2 - FpSGAT overexpressors line 2
L3 - FpSGAT overexpressors line 3
